# Supplementary material for: Causal interplay between lactose intolerance and gut microbiota: a combined bidirectional Mendelian randomization and in vivo validation study
Source: Front Nutr. 2026 Jun 1;13:1803337. doi: 10.3389/fnut.2026.1803337 (PMC13265576; doi:10.3389/fnut.2026.1803337)
Supplement: Supplementary file 1 [file Data_Sheet_1.zip › supplementary materials/Forward/leave one out cross validation/ebi-a-GCST90027812.finngen_R12_E4_LACTONAS.pdf]

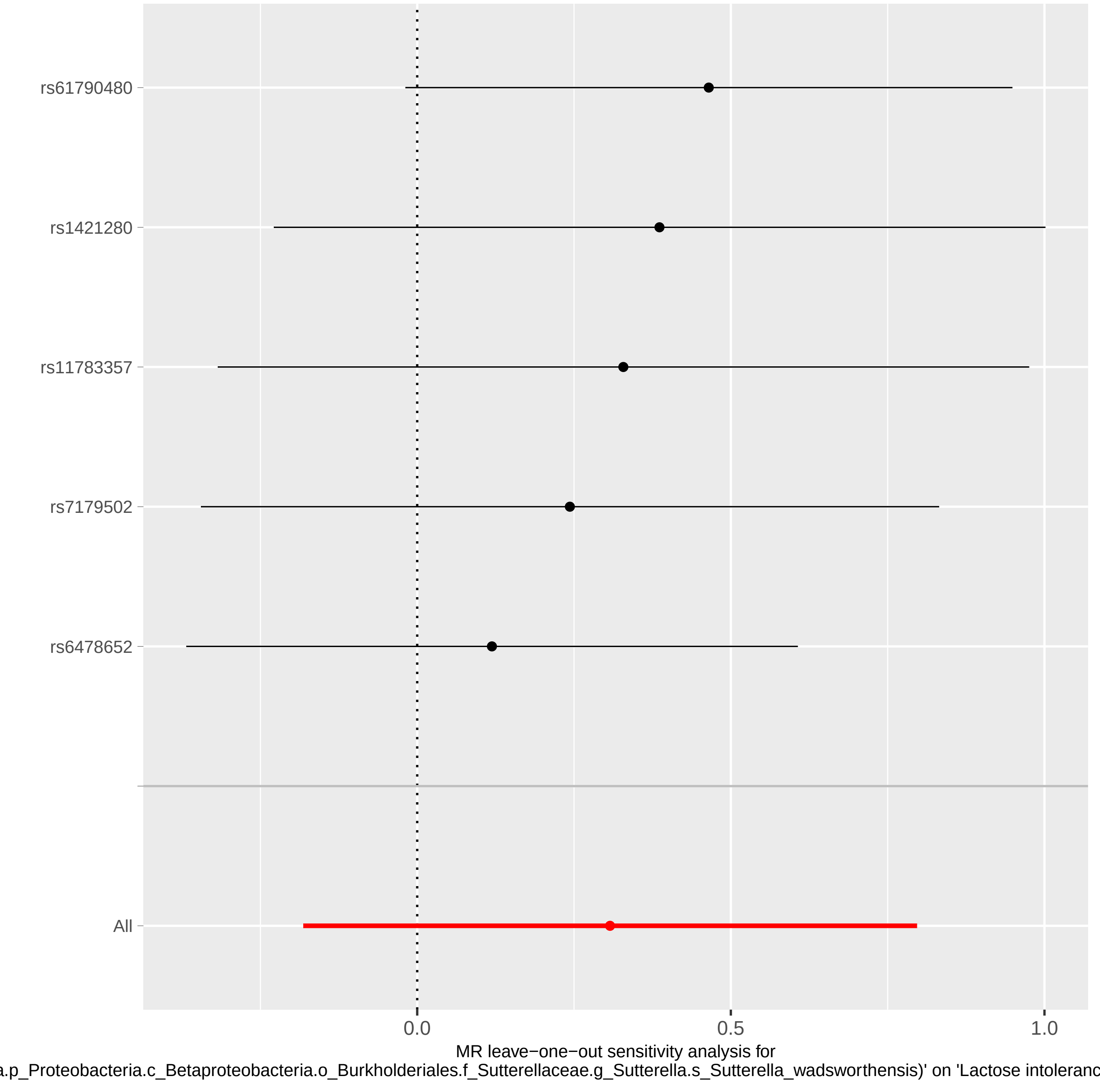
rs61790480
rs1421280
rs11783357
rs7179502
rs6478652
All
0.0
0.5
1.0
MR leave−one−out sensitivity analysis for
a.p_Proteobacteria.c_Betaproteobacteria.o_Burkholderiales.f_Sutterellaceae.g_Sutterella.s_Sutterella_wadsworthensis)' on 'Lactose intoleranc
